# Teacher and Caregiver Perspectives on Water Is K’é: An Early Child Education Program to Promote Healthy Beverages among Navajo Children

**DOI:** 10.3390/ijerph20176696

**Published:** 2023-08-31

**Authors:** Carmella B. Kahn, Brianna John, Sonya S. Shin, Rachel Whitman, Asia Soleil Yazzie, Renee Goldtooth-Halwood, Ken Hecht, Christina Hecht, Laura Vollmer, Malyssa Egge, Nora Nelson, Kerlissa Bitah, Carmen George

**Affiliations:** 1 College of Population Health, University of New Mexico, Albuquerque, NM 87131, USA; 2 Division of Global Health Equity, Brigham and Women’s Hospital, Boston, MA 02115, USA; 3 Community Outreach and Patient Empowerment Program, Gallup, NM 87301, USA; 4 Notah Begay III Foundation, Albuquerque, NM 87004, USA; 5 Nutrition Policy Institute, Division of Agriculture and Natural Resources, University of California, Oakland, CA 94607, USA; 6 Cooperative Extension, Division of Agriculture and Natural Resources, University of California, Davis, CA 95618, USA; 7 Community Member, Bluff, UT 87512, USA; 8 DigDeep, Los Angeles, CA 90021, USA; 9 T’iis Nazbas Community School, Teec Nos Pos, AZ 86514, USA

**Keywords:** Indigenous, Navajo, American Indian, early child health, water, sugar-sweetened beverages, community-based participatory research, early child education

## Abstract

The Water is K’é program was developed to increase water consumption and decrease consumption of sugar-sweetened beverages for young children and caregivers. The pilot program was successfully delivered by three Family and Child Education (FACE) programs on the Navajo Nation using a culturally centered curriculum between 2020 to 2022. The purpose of this research was to understand teacher and caregiver perspectives of program feasibility, acceptability, impact, and other factors influencing beverage behaviors due to the pilot program. Nine caregivers and teachers were interviewed between June 2022 and December 2022, and a study team of four, including three who self-identified as Navajo, analyzed the data using inductive thematic analysis and consensus building to agree on codes. Five themes emerged, including feasibility, acceptability, impact, suggestions for future use of the program, and external factors that influenced water consumption. The analysis showed stakeholders’ strong approval for continuing the program based on impact and acceptability, and identified factors that promote the program and barriers that can be addressed to make the program sustainable. Overall, the Water is K’é program and staff overcame many challenges during the COVID-19 pandemic to support healthy behavior change that had a rippled influence among children, caregivers, teachers, and many others.

## 1. Introduction

Access to safe and appealing drinking water is essential for early child health [1]. Adequate hydration is important for health; however, it is important that hydration be from healthy sources. Plain water is a healthy substitute for sugar-sweetened beverages (SSBs), which are a risk factor for many chronic conditions, including cardiovascular diseases, cancer, Type 2 diabetes, dental decay, and obesity [2]. These health conditions disproportionately impact communities of color, in large part due to social determinants, such as low access to healthy food and drinking water, that shape health disparities [3]. This is particularly true for Indigenous communities such as American Indians and Alaska Natives (AIANs) in which high rates of diet-related health conditions are well-documented [4]. For this reason, pediatric experts recommend children under age six drink only water and milk and avoid SSBs [5]. 

Water has strong cultural ties to the Diné (Navajo) people, who live on the Navajo Nation, an area of 27,425 square miles located in the southwestern high desert region of the United States [6]. Water is thought of as a living entity which should be protected and shown respect as it helps to preserve the livelihood of the Diné [7]. Due to historical exploitation of natural resources and underinvestment in water infrastructure, the Navajo Nation continues to deal with issues of water contamination and scarcity, including lack of running water in approximately 30% of households on the rural reservation [8]. This means many families must source water outside the home, often hauling water or purchasing bottled water—both of which are associated with excess travel and financial and opportunity cost.

### 1.1. Past Programs Promoting Water Consumption among AIAN Communities

Only a few programs have been implemented in AIAN communities to tackle the issue of promoting water over SSBs for young Indigenous children. Interestingly, these programs approached water promotion with different goals, ranging from enhancing environmental health literacy to preventing obesity. LaVeaux et al. [9] developed a water curriculum, “Guardians of the Living Water”, to increase environmental health literacy among 9-to-11-year-old children in the Crow community. The curriculum, delivered in summer camp and after-school sessions, shared tribal stories and knowledge along with local water quality information to cultivate interest, understanding, and skills among children to understand their role in protecting water in their community. Participant surveys demonstrated significant increases in environmental health literacy among participating children and their families [10]. Qualitative findings highlighted key takeaways for both children and caregivers that included water as a sacred element, how to understand water quality, and the importance of water conservation. Another program, Family Spirit Nurture, was developed in 2019 by Johns Hopkins University in partnership with tribal communities to address feeding practices in infancy to reduce childhood obesity, using culturally informed information [11]. Among the topics covered, “Rethink That Drink” provided information to understand the infant health effects of SSBs. Family Spirit Nurture resulted in significantly less consumption of SSBs among year-old infants (0.56 cups in the intervention versus 1.78 cups in the control group, *p* < 0.001) [12]. The Notah Begay III Foundation has taken a non-research approach, developing culturally relevant resources and an App to promote water and supporting community-led initiatives across diverse Indigenous communities [13,14,15].

### 1.2. Cultrally Centered Health Promotion

These initiatives highlight the importance of community input and incorporating culture and tradition into the content. Culturally entered health promotion has proven to be successful in addressing health needs of Indigenous communities [16]. This involves listening and working with Indigenous communities so that their voices and opinions can be amplified when addressing the health concern [17]. By using community-based health practices, researchers can work closely with Indigenous communities to deliver critical health information. For example, in many Indigenous families and communities, the role of the elder is important as they are often held in the highest regard. They often help with delivering culture and traditional knowledge to younger generations [18]. Incorporating input from elders and ensuring that community experts are respectfully acknowledged and compensated for their time is regarded as a best-practice for community-based participatory research (CBPR) [19].

### 1.3. History of the Water Is K’é Program

Our team underwent a similar journey in developing “Water is K’é”, a culturally centered intervention to promote water and early child health, delivered by early child education teachers to children ages two to five and their caregivers on Navajo Nation [20]. Working closely with a Community Advisory Group (CAG) comprised of teachers, elders, caregivers, health promotion experts, activists, students, and healthcare providers, we designed an intervention using three core strategies to promote behavior change: cultural connection (the role of water based on Diné language and traditions), health literacy (understanding the effects of water and SSBs on child health and how to identify SSBs), and water access (promoting strategies in homes and early child education centers to maximize children’s access to safe and palatable water). From 2021 to 2022, six Family and Children Education (FACE) programs located on Navajo Nation took part in Water is K’é, using the lesson plans with children and their primary caregivers (such as parents or another close relative) enrolled in their schools. FACE is a national program run by the Bureau of Indian Education that serves American Indian families from pregnancy to five years of age by providing early childhood, parenting, and adult education services [21].

In addition to quantitative surveys, we felt it was important to gather perspectives from participating teachers and caregivers in order to understand their experience of our pilot intervention, Water is K’é. The purpose of this qualitative study was to understand stakeholder perspectives of Water is K’é, specifically with regards to program feasibility, acceptability, and impact, as well as understanding other factors that influenced their beverage behaviors and ways to improve the pilot. 

## 2. Materials and Methods

The Water is K’é program was funded by the Robert Wood Johnson Foundation Healthy Eating Research grant between 2020 to 2022. The Navajo Nation Human Research Review Board (NNR-21.409) and Partners Institutional Review Board (IRB) (2020P001537) approved the Water is K’é program and the data collection activities for the interviews and focus group. Water is K’é consisted of four lesson plans, delivered either in homes or at the schools over the course of four months (one lesson per month). Each lesson plan included a caregiver session (which shared information and parenting tips to promote healthy beverage consumption) and an “activities” session for children and caregivers together (which encouraged choosing water over sugary drinks through Diné culture and language, play, and art). In addition to activities and hand-outs, families received infused water recipes (i.e., flavoring plain water with fresh fruit and no other additives) and “Potter the Otter” books [22]. In hopes of reducing plastic waste, the project provided high-quality reusable water bottles for each caregiver and child. Collective goal setting by the caregiver and child was encouraged in the form of a monthly “family promise”. Families were encouraged to choose sources of water based on their preference and safety perceptions rather than the project providing water as a component of the intervention.

The Water is K’é program continued even during the COVID-19 pandemic. The emergence of COVID-19 on the Navajo Nation signaled to officials that an emergency administrative order needed to be issued. The administrative order, which included restricted travel, limited capacity in public spaces, and limited establishment hours, was issued in April 2020 by the Navajo Nation. The administrative order also included the closure of in-person classes, forcing educators to navigate and adapt their lessons for an online platform. 

Due to COVID-19, in the first year, all activities took place in caregivers’ homes, with teachers facilitating sessions by phone, Zoom, or WebEx. In the second year, activities took place on-site at schools, usually through hybrid learning, allowing families to take part in person or remotely through Zoom or WebEx. The lesson plans were developed for use both online and in in-person delivery, including monthly packets containing all the materials (e.g., handouts, coloring pages, crayons, or paints) needed. For remote learning, packets were delivered before the online class; for in-person learning, they were handed out at the beginning of class.

### 2.1. Interview Guide

The interview guide was drafted by study staff and then shared with the CAG for feedback. The CAG suggested several additional questions to understand external factors shaping health behaviors (e.g., other programs, COVID-19) and improved the wording of questions for better understanding. The final guide consisted of six main questions and sixteen sub-questions related to Water is K’é implementation, behavioral outcomes, and challenges and strategies for children to drink more water. Examples of questions included: (1) Were there any ways that Water is K’é was hard to implement? Please describe. (2) Could you describe any changes in kids’ behaviors that you have observed as a result of this program? (3) What do you think are the most effective ways to get children to drink healthier drinks? The CAG also added the question, What other programs did they take part in? The full interview guide is included in Appendix A. 

### 2.2. Recruitment and Data Collection

All parents/caregivers lived on the Navajo Nation and took part in the Water is K’é program in the 2021–2022 academic school year. Caregivers were recruited on the day they completed their exit survey for qualitative interviews. As the caregiver handed in their paper forms, our team explained the purpose of the interview and invited them to sign up if interested. The study staff then made follow-up phone calls, text messages, and emails to schedule interviews based on availability. Several caregivers signed up to participate in the interviews; however, only a total of three parents were able to complete the individual interviews. Interviews were completed in June 2022 through Zoom by four study staff and lasted between 20 to 40 min. 

Teacher participants were recruited from early child education schools who administered Water is K’é in the spring of 2022. The study staff emailed the schools to recruit and share information about the purpose of the interviews or focus group. At each site, all teachers involved in delivering the program were invited to take part in an interview, which could be individual or group, in person or virtual, depending on teachers’ preferences. Teachers who agreed to participate had previously completed a consent form when they were recruited to take part in the Water is K’é program. Of the three Family and Child Education (FACE) program sites that took part in spring 2022, two schools agreed to participate, while the third could not be reached due to staff turnover. Teacher interviews took place in December 2022 in person and via Zoom and lasted 30 to 75 min. 

All interviewers self-identified as Navajo. To reduce desirability bias, we hired a research consultant who had not worked directly with teachers to conduct those interviews. Interviewers were first trained how to use the guide, including ways to probe based on the participant group (caregivers versus teachers). Before beginning each interview, verbal consent for the interview and audio recording was obtained. Interviews were recorded using a digital recorder or Zoom, and then downloaded and saved. Caregivers and teachers who participated also received a USD 25 gift card for their time. All interviews were completed in English and data were transcribed by the study team, using pseudonyms for names and places. English was the preferred language for all participants. 

### 2.3. Data Analysis

The data analysis was completed with Microsoft Excel by a team of four researchers (CBK, SSS, BJ, CG), including three who self-identified as Navajo. One study staff member (CBK) drafted a code book, identifying codes and definitions through inductive thematic analysis based on teacher interviews. Another study staff member (SSS) edited the code book based on caregiver interviews, added or modifying themes, sub-themes, and definitions as needed. The team then met to review the draft and arrive at semi-final codes and definitions. One researcher (CBK) used the new code book to code all parent transcripts, while another researcher (BJ) coded 30% of all transcripts. The study team met to compare double-coded content and reach a consensus on the final coding. During this time, a few codes were modified, resulting in a final code book for final coding of all interviews (CBK). One study staff member (CBK) wrote summaries from a combined analysis of the caregiver and teacher codes. Based on the summaries, five themes and associated patterns were identified from recurring or key concepts: feasibility (i.e., how doable was it to implement the program), acceptability (i.e., did participants like the program; what did they like?), impact (i.e., change in behavior, knowledge, and environment); suggestions for the program; and external factors (barriers and promoters influencing water consumption). The results were presented to the CAG and their feedback was incorporated into the interpretation of findings. 

## 3. Results

A total of nine (*n* = 9) adult participants completed the interviews. The study team completed three individual Zoom interviews with caregivers, one individual in-person interview with a teacher, and one group interview with five teachers via Zoom. The pool of participants included three female parents, one male teacher, and five female teachers. Five themes were identified, including: (1) feasibility, (2) acceptability, (3) impact, (4) suggestions for future use, and (5) external factors influencing water consumption. 

### 3.1. Program Feasibility

#### 3.1.1. Overall Feasibility

In terms of overall feasibility, caregivers and teachers generally felt that it was feasible to implement the Water is K’é curriculum in their classrooms and homes. Caregivers indicated they did not face any challenges being in the program and felt the pre-assembled packets were understandable and easy to complete. Teachers also mentioned there was no challenge implementing the program, particularly if they had the support of the principal and if they prepped in advance by reading ahead. 

#### 3.1.2. Facilitators and Barriers to Program Initiation

Several factors contributed to successful initial engagement, including recruitment efforts and having administrative support. Teachers felt the proactive efforts by the study team (RW, ASY, CG) helped to build interest and buy-in for school participation. This included multiple recruitment emails, as well as in-person outreach to explain the study and program, such as a recruitment presentation and sharing sample program topics related to cultural teachings. As a result, five FACE schools were successfully recruited, even at the height of the COVID-19 pandemic. For one FACE site, program implementation and continuity depended on the interest and approval from the principal.

As programs got underway, several facilitators and barriers were mentioned. Initially, teachers did not know what to expect with the lesson plans, since materials were shared in phases. Rather than provide all lesson plans at once, the study team decided to distribute packets month-by-month, allowing the team to go over that month’s lesson plan at the beginning of each month. While this helped reduce confusion regarding which lesson plan to use, some teachers expressed uncertainty because they did not know which lesson was “coming next”. Over time, however, they had the complete curriculum and knew what to teach. Initial apprehensions were also relieved once they realized that prep-time was minimal due to prepackaged lesson plans, making it feasible to deliver the sessions without excessive demands on time. Another approach that helped with planning and time management was working as a team. In one school, teachers decided to work as a team, taking turns delivering lessons and assigning a team leader. Teachers were happy to share responsibilities and were grateful that they could rely on a team leader to guide them on future lessons and answer any questions.

*[The team leader] kept us on track. So, she was our go to person. I know that if I got lost, I just went to her… I’m kind of glad each one of us got to do it… If I had to do the whole curriculum [on my own], … that wouldn’t be good. So, Teacher 1 really did help us.*

(Teacher, female)

#### 3.1.3. Adapting to Modes of Delivery

Many of the barriers to and promoters of program feasibility related to adapting to different modalities (i.e., home-based and site-based/hybrid) due to pandemic restrictions. In the first year, remote learning presented challenges related to Zoom, technology, and Internet access. One teacher stated that her school switched to a basic Zoom plan, allowing only 40 min per session. As a result, she felt rushed during her lessons. She observed it was important for schools to pay for a Zoom plan, so they could hold lessons without time pressure, and while she would like to continue Water is K’é in future years, she felt feasibility would depend on whether classes would be conducted in person or remotely by Zoom. Another teacher said she was not able to use some of the materials online, commenting that she felt that the materials were easier to use in person. For caregivers, similar issues of unreliable Internet, limited access to technology (i.e., access to good cell phones or computers), and phone issues (such as having limited minutes on their plans) introduced challenges.

*[If] our internet went out, then that was a challenge, too. So, if it slowed down or, you know, things like that… and then parents…gosh, I’m trying to remember last year, they didn’t have any devices. But you know what, these parents, the parents we had last year, they actually got on with whatever devices they had…their phones, or whatever they will participate.*

(Teacher, female)

Despite these challenges (or because of them), teachers and caregivers found creative ways to participate using whatever technology was available to them. Teachers came up with creative strategies to adapt to online teaching. Since families needed to receive a lesson plan packet each month, teachers worked with FACE bus drivers to deliver the packets before each session since teachers themselves were not allowed to visit homes. In that way, caregivers had the packets before each monthly Zoom session.

*Last year, being virtual and remote learning, it was kind of hard, but our bus drivers delivered all of our learning packets. So, we just put in the stuff from the K’é project, and put it in the learning packet, and they’re the ones that delivered it for us, so the drivers really helped us out last year.*

(Teacher, female)

Maintaining engagement and interest online was also a hurdle, often because of competing responsibilities at home. For some caregivers, it was difficult to complete the lesson plans or attend every session. One parent mentioned she had a hard time completing some packets because of her work schedule. Another parent mentioned they only listened to the first few lessons and had a hard time recalling some of the lessons. Again, teachers and caregivers came up with imaginative solutions to this problem. One school used a parent agreement to concentrate on the lessons by turning off other electronic devices. Another teacher asked caregivers to log in on time and have learning packets on hand to access them easily. During the lessons, teachers engaged caregivers by asking them questions to increase participation. Caregivers also found ways to overcome remote barriers. Sometimes, caregivers conducted the lessons in front of the teachers, and, in one example, a parent had her children get in front of the camera to participate.

As schools shifted back to optional site-based learning, caregivers were given the option to participate in person or remotely. Most families opted to join by Zoom if they had the technology; otherwise, they would go in person to the FACE classroom. Teachers observed that the quality of the delivery and content was similar to that of Zoom lessons. In this sense, teachers felt it was feasible to shift Water is K’é from remote to site-based delivery. While the hybrid approach offered more flexibility for families, some teachers observed that this required extra work on their part. One teacher felt that virtual lessons were more manageable because they still had to deliver in-person lessons in front of a computer for those who were on Zoom. This required extra preparation to conduct hybrid lessons, including sharing information and videos in advance. Nonetheless, teachers went above and beyond to deliver the program, such as breaking up the lessons into quick conversations during brief interactions when the caregiver would come in to pick up their child.

*I’ll have to be a little bit more creative … they’ll pick up their kids here at this school sometimes, and I’ll go out there and I’ll talk, you know, do a visit with them really fast. And, you know, I’ll do it by phone also. So, I’ve been having to do a lot of…you know what I mean, running around.*

(Teacher, female)

It is worth mentioning that FACE programs also have home-based services where teachers work one-on-one with families in their homes. Because of COVID-19, teachers were not working in the homes, so the program’s feasibility in this setting was not tested. However, when asked, teachers felt that it would not be a problem to deliver the lessons one-on-one in families’ homes.

### 3.2. Program Acceptability

#### 3.2.1. Overall Program Acceptability

Overall, the program acceptability was high, as evidenced by high rates of participation and interest in continuing the program. A teacher mentioned that the majority of families were consistent with their participation because they felt COVID-19 was serious and they needed to stay healthy.

*But… they knew the seriousness because of COVID, you know, that staying healthy was really important. And so…they really did take note of that. And…these are the ones that were consistent with their participation, which was really the majority of them.*

(Teacher, female)

Caregivers indicated they would like to continue with the program, especially if new material was added. Teachers also expressed they would like to continue with the program and felt that offering the program in future years could reach new families, including younger parents, who could benefit from the program.

#### 3.2.2. Promoting Diné Culture

One of the program’s greatest appeals was its emphasis on Diné culture. Both teachers and caregivers commented that the cultural features of Water is K’é motivated them to join the program. After doing the lessons, they consistently mentioned how much they appreciated the cultural elements, such as Diné teachings that water is medicine and how water clans are significant (Diné have a kinship system based on clan groupings). Another positive feature was the video with Diné teachings from elders about the sacredness of water. Caregivers enjoyed being reminded about the sacred ways of water and how it can heal. One caregiver mentioned she, in turn, helped her daughter understand these teachings.

*When I was talking to Miss “Begay”, she was telling me that water is sacred, and she wanted the kids to understand that water is sacred for us, and that it can heal us. And it can make us grow taller and stuff. Like she was basically saying that to [my daughter] when I was asking her about that, like what made you guys want to do this program. And that’s what she said to me, and I like that they did.*

(Parent, female)

#### 3.2.3. Engaging Multiple Generations

Another strength of the program was how it engaged multiple generations. One teacher said the families were engaged and answering questions, and the kids participated in the activities and liked the stories. Teachers appreciated how lesson plans were designed so caregivers and children could learn together. One teacher even mentioned that grandparents listened to the sessions along with the parent and child. Caregivers said their children liked the hands-on activities and items, such as the free reusable water bottles, using the program coloring book, and learning Navajo words about water. A teacher also commented that families liked how the reusable water bottles could be personalized with stickers. Another parent said her son particularly enjoyed the infused water with strawberries. Caregivers also felt they learned a lot about the importance of water and how much water they are supposed to drink. 

#### 3.2.4. Alignment with FACE Program Services

Water is K’é was also viewed as an appropriate curriculum to meet their goals as an early child education program. One teacher mentioned they were always eager to learn more and offer new programs to their families; their school had taken part in other programs and studies, and was interested in Water is K’é as an opportunity to teach new information to their families. One teacher expressed her own personal motivation to drink water, and appreciated how the program could benefit their families.

*But I think that our program is also, you know, we have been involved in a lot of…different types of programs, or studies…I think naturally we just knew that…we can participate and see how this would help our parents, because we’re always trying to find ways to bring new information to our parents.*

(Teacher, female)

### 3.3. Program Impact

The program impact was observed through its influence on changes in knowledge and behavior among caregivers, children, and teachers. Caregivers and teachers also shared different strategies that they incorporated to make healthy changes as a result of the program. 

#### 3.3.1. More Knowledge about Health and Diné Traditions

The program influenced caregiver’s knowledge about ways to drink more water. One parent mentioned she gained knowledge in learning to drink more water than little sips throughout the day based on the recommended daily intake listed on the lesson packet.

*The only thing that I noticed that we were changing and that we were learning is that we need to drink more water instead of just drinking half a cup a day, or just little sips here and there. And when I was looking at the package, I realized how much water that we are supposed to take daily, and I was telling my husband, I was like, ‘I never knew we’re supposed to drink this much water!’*

(Parent, female)

Families also gained knowledge about Diné language and culture. Caregivers liked learning the Diné words and traditional teachings about water. Another parent felt the lessons were interesting and got her thinking a little more about traditional teachings. The sessions also helped remind caregivers about traditional beliefs about water and why it is good to drink water, and they remembered teachings they had growing up. Teachers also observed that caregivers learned about how water is used in Diné culture and were using some of the Navajo language terms at home like ‘dooda soda’ (no soda). 

The program influenced children’s knowledge to purchase and consume water. One child gained new knowledge about water and became a conscious shopper, wondering why different brands of water bottles all look the same. Another child enjoyed taking learning packets home and sharing what he learned with this family. One parent shared that their kids were learning about traditional beliefs about water and why it is good to drink water.

The teachers also shared how the program influenced their own knowledge about the importance of water both in relation to culture and health, such as how clans are related to water and have water within the clan names, why water is the healthiest drink, and how much water is in our bodies.

*Water is the only thing that’s probably good for us. But I don’t think there’s anything called, any such thing as a healthier drink. Every drink has a lot of sodium, every drink has a lot of sugar, even though it says no sugar.*

(Teacher, female)

#### 3.3.2. Healthier Beverage Habits

Caregivers were influenced to drink more water and fewer sugary drinks. One parent mentioned seeing the handout about sugar intake was an eye opener and helped her stop drinking soda; as a result, she felt more energetic and less sleepy and healthier overall. Another parent expressed a challenge initiating behavioral changes after doing the lessons. She described her family’s effort to consistently drink water and not drink sugary beverages. Initially, the changes were hard for the caregivers, but once they initiated new drinking habits and overcame the cravings, they felt it was better for them. Teachers also observed that caregivers were drinking more water, monitoring their sugary beverage intake, and understanding how it affected their health overall. They felt that activities such as putting up posters at home and using water bottles helped facilitate these changes. Teacher tips, such as encouraging parents to drink more water, also helped some parents make changes.

*We drink more water. We got rid of sodas. We don’t drink soda no more, like we stopped and maybe the only thing we drink is water, juice pouches and Gatorade. We’d been talking about getting rid of sodas, but after we saw the [handout], like how much sugar we intake with sodas, and we just ended up stopping and that’s the one that was a big change and was hard for us to do.*

(Parent, female)

Caregivers and teachers felt that Water is K’é helped their children drink more water and less sugary drinks. One parent commented her daughter preferred water and became particularly interested in one of the coloring sheets that showed how many glasses of water she should drink, even reminding her parents to drink water as well.

*[My daughter] was very curious about where water comes from, and she looks at the chart [coloring sheet]… and she’s very curious of how much water she’s supposed to be intaking. And sometimes, when we don’t [drink enough water], she kind of gets mad at us for it. So, she has a little schedule going about drinking water. And that’s a big change for us, because she’d never done that.*

(Parent, female)

Teachers also observed the same changes as children began to drink more water and even remind their parents to drink water. One teacher said they heard children say “soda dooda” (no soda) to their caregivers if they were not drinking water.

*One of the things we always heard them say is soda dooda. The kids were saying that. The kids were even saying that with their parents. They would see their parents have something other than water around them… they would just go home and say soda dooda.*

(Teacher, female)

In terms of Water is K’é materials that really helped, both teachers and parents mentioned infused water recipes were helpful in getting children to drink more water because they liked cutting up fruit, tasting, and getting creative with flavors. One participant mentioned that the Potter the Otter books were a nice complement, showing children how to add fruit to their water. The water bottles were also used regularly at home. For caregivers, it was useful to see the handout on the amount of sugar in soda and recommendations for daily water intake based on age. 

Interestingly, teachers were motivated by Water is K’é to make changes in their own beverage choices and encourage healthier habits, both in and out of the classroom. One teacher commented how the program influenced her to encourage her grandchild to drink more water. Another teacher commented that she encouraged and reminded her adult son to drink more water. One teacher mentioned they provided water for in-person family circles. Other teachers shared how they encouraged students to drink water after doing physical activity and have it readily available.

*And then we encourage them to like have water after like outdoor, or physical when we do some physical activities. After that we encourage them to drink water. So, we do encourage, you know, the kids to drink water and then having water readily available.*

(Teacher, female)

#### 3.3.3. Sharing Strategies to Promote Healthy Beverages 

Beyond the Water is K’é program, many participants already had successful strategies to increase water and reduce SSB consumption. Several families shared how they felt confident with their water quality. One parent got their water tested for lead and felt more confident drinking it after it was found to be safe to consume. Another family had a filtered water dispenser in their fridge, so the family felt their water was both safe and refreshing.

*We have filtered water, and our refrigerator has a filtered water spout with the ice maker as well. So, we actually really don’t have much of a problem with drinking water. It’s always refreshing, especially when you get some ice in there.*

(Parent, female)

None of the participating FACE sites had soda machines and all only offered milk or water during meals and bottled water in the classrooms. As part of their health screening, teachers include a question about the types of beverages that caregivers give to their children. One teacher mentioned that these policies were important to promote healthy beverage environments, and encouraged her families to do that at home. In fact, at least one family practiced this strategy, choosing not to have sugary drinks in the house so there is no temptation to drink sugary beverages.

*We just try not to have any sugar drinks available or in the house, for him to have that urge to drink sugary drinks. So all we have is just that 100% juice or some water or milk.*

(Parent, female)

### 3.4. Suggestions for Program Improvements

Suggestions for future use of the program included feedback on curriculum changes, additional activities, and expanding the target group. Overall, caregivers felt the curriculum was complete, although several felt that free water testing and more program promotion (on Facebook and flyers) could help. While some teachers did not think any changes were needed, several teachers had creative ideas. For instance, one teacher suggested using a cartoon character (“like one from Space Jam”) to describe how water is like a miracle or magic in order to appeal to children to drink water. Other teachers suggested questions they could use to expand on discussions with caregivers around the topics of health and culture. Other ideas included more books about drinking water and healthy beverages, gift cards to bookstores, and “Water is Medicine” T-shirts. 

In terms of target group, one teacher suggested involving other caregivers such as grandparents, since they were also responsible for children’s beverage habits. Teachers also suggested offering the curriculum to pregnant mothers and mothers with newborn babies, and expanding the program to more schools and teachers in order to influence more students about the importance of water and cultural aspects. Another teacher felt the curriculum could be shared with other FACE programs across the US, not just Navajo Nation.

*I know the curriculum is only for a certain age… but the best time to really get some of these parents is when they’re either… during prenatal and when their babies are newborn. You know, I think that it’s gotta go down lower because… just making them aware that… being healthy during pregnancy and you’re doing this for the baby, and you’re drinking a lot of sugary stuff, and… what is that doing to the baby?*

(Teacher, female)

### 3.5. External Factors Influencing Water Consumption

The Water is K’é program was piloted during the pandemic, which influenced external factors that also helped and hindered the ability for caregivers to choose healthy beverages for themselves and their children’s decisions and behaviors. 

#### 3.5.1. COVID-19 and Economic Hardship

Interestingly, COVID-19 and economic factors had both positive and negative influences on beverage behaviors. On one hand, COVID-19 lockdowns made it harder to buy water and fruits (for infused water) because caregivers had to shop quickly or less frequently, and fresh fruit was also often out of stock.

*Yeah, in fact, it did because we weren’t able to get any of the fruits for the flavor water. It was kinda really hard to go to town and pick out the stuff that we needed. Because it’s kind of like we have to hurry, hurry, hurry to get in there and get out. Cause like it’s only a minimum of people can go in, and sometimes when we do that, it will be like, just go in and get out.*

(Parent, female)

On the other hand, because families were home and shopped less, some of them began to eat three balanced meals at home and less snacks or junk food.

*Because we really had to start considering especially at the time when it was the worst when we couldn’t go out and go shopping. We really had to be creative and consider, you know, finishing all our meals. And there was no snack time, junk food, it was more of a balanced 3-day meal. I know that’s one thing that happened during the pandemic.*

(Parent, female)

Teachers also described how schools suspended access to water fountains in an effort to reduce COVID-19 spread, providing pre-packaged bottled water instead. At the same time, teachers shared with caregivers why drinking water was important to stay healthy during the pandemic, which may have promoted healthier behaviors.

In a similar way, economic hardship posed both a challenge and advantage for water consumption due to changes in family income and rising prices. Families mentioned water prices had increased; one parent mentioned that a filtering system at home could be a solution as cases of bottled water got more expensive. Teachers noted that many caregivers were struggling with costs and not having jobs and they were staying home more often. They speculated that some families were buying more water because soda was expensive.

#### 3.5.2. Beverage Marketing Is Everywhere

Marketing was also a challenge because it was a source of confusion and temptation with gimmicky packaging and advertising for kids. One parent commented that her daughter gets confused when choosing water to buy because they all seem the same and its challenging to know which ones are healthy. Another parent mentioned their child sees fancy juice bottles with characters like action figures on the package, and they end up buying that drink for their child. One teacher noted that advertisements are everywhere; big box stores make it tempting to buy sugary beverages by offering free samples, and convenience stores offer more sugary beverage choices than water. Even at home, TV advertisements influence families to choose drinks besides water.

*As far as their advertising… most of our families have TV. So, that kind of influences them because I do come across a lot of my families that do… want to choose other drinks than water.*

(Teacher, female)

#### 3.5.3. Caregivers Are Already Knowledgeable

Many caregivers were already quite knowledgeable about healthy beverages and child health, through various outlets of knowledge sharing and skill building. Many families had already received information from health centers and tribal health programs about making healthy drinks with water, understanding SSBs, and how to read labels. One health center offered free water lead testing. FACE programs incorporated health into their programs, inviting health promotion partners to share information and promoting water through other resources, such as a book called *Water Protectors* to understand traditional perspectives of water. During the pandemic, another teacher in the school was also instrumental in sharing his knowledge about water and helping staff stay active.

As a result, many families shared that they already had healthy habits. One parent said his child already drank only water and pure fruit juice, even before they started the program. One teacher mentioned she and her family already drank only water before the program.

#### 3.5.4. Trusted Water Sources

The type of water source was also a positive factor to increase water consumption if it was deemed safe, accessible, and tasted good. As described above, some families took it upon themselves to test their home water or use a filtered system. Another family felt comfortable and confident drinking from their tap. At schools, bottled water provided the greatest reassurance of safety and palatability. Sometimes water confidence was at the community level, rather than the household level. Teachers at one school mentioned the tap water in their community was pristine; in fact, relatives traveled to their community to fill up their bottles for personal use or bought water from the chapter house for their livestock. While most families talked about the importance of access to running water in their homes, another consideration was plumbing. Despite lessons to drink more water, families with outhouses might limit their water intake so they do not have to use the outhouse so often.

#### 3.5.5. Intergenerational Role Models

Many caregivers and teachers talked about the importance of role models in the family; adults and children served as role models for each other to drink more water and less SSBs. Teachers and parents discussed how caregivers should be mindful about what they drink and what they offer to children. One teacher commented that the caregivers seemed to drink more water because they wanted to be role models for their children. As previously mentioned, children were often not shy about calling out their parents when they were drinking soda or not drinking enough water.

*I think that you could influence more children if you influence the teachers… the adults, because, you know, I’m pretty sure… although they would like to, they don’t drink as much water or they don’t have the knowledge as to why water is so important.*

(Teacher, female)

## 4. Discussion

### 4.1. Key Lessons from Water Is K’é

This qualitative study revealed important perspectives from caregivers and teachers who took part in Water is K’é, a culturally grounded program for families enrolled in FACE programs on Navajo Nation. The main findings from these interviews demonstrated the strong potential for this health promotion program delivered by early child education staff to positively influence water intake and decrease SSB intake. Several key lessons emerged.

#### 4.1.1. Designing a User-Friendly Program

Even in the midst of COVID-19, Water is K’é was feasible and acceptable, due to evidence-based strategies that made the curriculum user-centered/friendly. The study staff strategically designed the program to be prepackaged so teachers did not have to spend a lot of prep time creating the lessons. The lesson packages included an organized teacher’s guide for each lesson and were hand delivered one lesson at a time so study staff would have a chance to check in with the teachers, answer questions, and build teacher confidence to deliver the lessons. Each lesson shared evidence-based information about water and SSBs, using simple language and activities that were fun for young children. Each lesson focused on one or two key points to avoid overwhelming caregivers and children with too many messages. Especially during COVID-19, the team had to make sure the program would not be too difficult or too long, so caregivers and children could add it to their day-to-day activities and not have too much homework. The staff also made the program family-based to help parents engage in activities with their children.

A similar program implemented during the pandemic used a similar approach to share a “boxed” curriculum that was user-friendly for American Indian parents and children 2–18 years old [23]. Their program was designed to limit stress on families and included fun activities such as arts and crafts and making healthy snacks. The program staff mailed their lessons and activity materials, including healthy snacks, in a box to each family. Additionally, the staff only communicated with one adult per family to limit the stress on parents. Another home-based health promotion program engaged parents and children two to five years old to focus on healthy eating and physical activity, including decreasing consumption of SSBs [24]. Their program included a mentor who taught lessons from a tool kit and engaged parents and children at the same time. Activities were designed to be fun, culturally acceptable, and easy to carry out each week. Water is K’é shares many user-friendly elements, reflecting important features that make health promotion programs feasible and acceptable to both caregivers and teachers. 

#### 4.1.2. Developing a Culturally Centered Curriculum

The caregivers and teachers expressed how the traditional teachings in Water is K’é were a motivation to take part and change their behavior. The study staff designed the program to focus on traditional teachings related to water, such as the Diné belief that water is medicine and a sacred entity to be respected. Water is K’é was developed with input from the CAG, who strongly emphasized the need to include personal and traditional stories from elders. They wanted teachings that shared how families survived when water was scarce, how they found water, and how SSBs were rare in the past. They felt it would be beneficial to share cultural stories about water that included the Diné deity First Woman and her pregnancy with the twin babies, how babies are surrounded by water, and how water is used in ceremonies for healing. Our findings demonstrate how utilizing a culturally centered strength-based curriculum can support behavior change among parents and children. Another study used cultural teachings and input from an advisory group consisting of American Indian community members and researchers [25]. Their support helped the team develop a culturally informed safety curriculum that considered community belief systems to ensure the curriculum did not offend participants. Similarly, the study by LaRowe et al. [24] included traditional food and teachings in their curriculum to support healthy eating among parents and children. The importance of culturally based interventions goes beyond Indigenous communities [26]. Qualitative studies involving parents of young children in both Latino and Black communities have highlighted the importance of cultural norms, upbringing, and taste preferences when designing healthy beverage interventions [27,28]. Ensuring that programs are culturally specific is a cornerstone for health equity strategies given minorities are disproportionately impacted by diet-related diseases [29].

#### 4.1.3. Behavior Change Ripples Out

Water is K’é was designed to motivate caregivers and children to make behavior changes. We found that caregivers and children influenced each other and other family members towards drinking healthier beverages. In addition, the program motivated teachers, who in turn influenced their family members and other individuals they interacted with. Overall, Water is K’é helped diffuse changes toward more water and less SSBs within the larger community, with potential to shift social norms over time. A similar approach to involve parents was used by Berns et al. [23]. The study team used newsletters to teach parents about child safety so they in turn could teach their children. The lessons were also designed to engage children with different interactive activities. Parental involvement in teaching children has also been used by another study relating to childhood obesity prevention [11]. Caregiver involvement is a cornerstone for effective interventions to reduce obesity and cardiovascular risk in children, particularly when the intervention targets both the caregiver and child and promotes behavior change strategies across socioenvironmental contexts (i.e., both in and outside the home) [30]. Our intervention reflects these best practices by engaging caregivers and children to facilitate changes not only in terms of health behaviors, but also to their home environments.

#### 4.1.4. Adapting during COVID-19

The COVID-19 pandemic occurred around the start of the program. Schools faced challenges to offer education, and families confronted issues of water and water access along with rising costs. It took flexibility and the input provided by CAG to achieve the program objectives. Teachers overcame many challenges to deliver the curriculum and encourage participation, all of which was a testament to their dedication and the flexibility built into the lesson plans. Study staff, teachers, and caregivers had to quickly adapt to changing modalities and implement innovative ways to stay connected despite the challenges with technology. Other American Indian health programs have also stressed the need for flexibility and creativity to address the limitations with Internet service and lack of access to laptops or cell phones during the pandemic [23,31]. Programs loaned computer equipment and hotspots to participants [31], switched in-person programs to Zoom [31], or may have decided to rely on delivering curriculum and materials by mail and staying connected only through phone calls [23]. While these adaptations were necessary during COVID-19, this flexibility will likely be helpful for program continuation beyond the pandemic. 

### 4.2. Limitations and Strengths

One limitation of the study was the small number of participants in the interviews, which may limit our ability to draw generalizable conclusions. We faced challenges organizing interviews, particularly because in-person interviews were not very feasible due to the pandemic. Zoom itself presented challenges if participants had challenges with audio or connectivity, or if participants were distracted by other things happening in the room (one parent had to rescue a loose hamster during the interview). 

In addition, participants that were interviewed may reflect selection bias, as those parents or schools may have had good experiences and were more eager to do an interview. For instance, the one school that was not interviewed (due to staff turnover) chose not to continue the program the following year. Their feedback would have been helpful to understand if they had any negative experiences. Given these are tight-knit communities and existing partnerships that study staff had with the FACE programs, there was potential for desirability bias. We tried to lessen this by selecting interviewers who had not worked directly with interview participants during the program.

The strengths of the study included having perspectives of both teachers and parents. Furthermore, the results showed consistency of the quality and type of experiences between teachers and parents who participated. Another strength was having study staff who were Native American, which could promote comfort and accessibility for participants who took part in the interviews. 

### 4.3. Future Research

Three recommendations for future research are suggested. The first recommendation is to complete an evaluation of Water is K’é to understand if quantitative data matched our qualitative findings in terms of impact on health behaviors and influence of traditional teachings. The second recommendation is to use the feedback from the stakeholders to improve the program, such as involving grandparents as suggested in the interviews. The third recommendation is to address the external factors that participants observed. While this was not within the immediate scope of Water is K’é, we recognize there are larger structural factors that compete with family-based interventions. The participants highlighted many external factors that influenced their behavior, the degree of trust and confidence in their water sources, and marketing of beverages to children. The results merit exploration of potential solutions to address the challenges from these external factors. While Water is K’é is appears to be feasible, acceptable, and impactful, behavioral change can only be equitably achieved and sustainable if policies, systems, and environmental factors are addressed to promote water consumption.

## 5. Conclusions

Water is a sacred and life-giving source that offers many health benefits. Our pilot intervention, Water is K’é, was designed around this fundamental concept to promote healthier behaviors based on culturally centered health promotion. Our qualitative study suggests that the pilot was feasible and acceptable, and identified core strengths of the program, as well as barriers that could be addressed in the future. Further research is needed to assess the program’s effectiveness. Lessons learned could be applied to other programs and policies to promote safe and equitable access to water in Indigenous communities. 

## Data Availability

As all Navajo research data collected belongs to the Navajo Nation, data presented in this study are available on request directed to the Navajo Nation Human Research Review Board.

## Appendix A

Stakeholder Focus Group Guide
Verbal Informed Consent
Thank you for meeting with me. This is a study to understand people’s experience in the Water is K’é program. As you know, Water is K’é is a project that promotes healthy beverages for Navajo children and the adults who care for them. We would like to talk with people like yourself to understand what it has been like to take part in Water is K’é. Taking part in this interview is totally up to you. If you agree to talk with me, our meeting will last between 60 and 90 min. You can skip any questions and you can stop the meeting at any time. Your answers are confidential, which means that I will not share your name or any personal information with anyone outside of our study team. None of your answers can be traced back to your name.
To thank you for your time with this focus group interview, I have a $25 gift card for you.
Please call us with any questions or concerns. Carmen George is the main contact for this study, and you can call her with questions at any time. Her phone is 505-860-7861.
Would you like to take part in this interview?
1. Why do you think [insert name of Head Start /FACE Program] decided to implement Water is K’é?
- Can you describe what changes your site made as part of the program?
- Can you describe any challenges in bringing Water is K’é to your site, and how you addressed these problems?
- Were there any changes introduced by Water is K’é that have been especially positive? Please describe.
- Were there any ways that Water is K’é created problems or was hard to implement? Please describe.
- Can you share any other campaigns or presentations that you participated in this past year that encouraged healthy beverage consumption?
- Can you share any traditional teachings that you listened to this past year?
2. Could you describe any changes in kids’ behaviors that you have observed as a result of this program? How about changes in adults’ behavior?
- What do ECE staff think about the program?
- What do parents and relatives think about the program?
- How about the kids? What do they think about Water is K’é?
3. After taking part in Water is K’é, has your site considered whether they plan to continue the program after this year?
- If yes, what challenges would you see in keeping the program running?
- If no, please describe why your site is not planning to keep the program going.
4. Healthy beverage habits includes drinking more water and drinking less sugary drinks.
- Can you share a little about what children are drinking when they are at home and in the community?
- Could you share what challenges you and your children face to drinking more water?
- Could you also share what challenges you and your children face to drinking less sugary drinks?
- What do you think are the most effective ways to get children to drink healthier drinks?
- How can we impact children to choose healthy beverages?
5. Are there any other comments or suggestions for improving Water is K’é? Anything else you’d like to share?
6. We know the past few years have been difficult on many levels. Are there any impacts that you have felt in making healthy choices? [Probe: COVID, Inflation, for children beverages or for healthy beverages]
Thank you very much for your participation!
